# The Anatomist's Vade Mecum; a System of Human Anatomy

**Published:** 1842-07

**Authors:** 


Art. X.—*The Anatomist's Vade Mecum; a System of Human Anatomy.* By Erasmus Wilson. Second Edition.—*London,* 1842. 8vo, pp. 595. With 167 Illustrations by Bagg.

On a former occasion (Br. & For. Med. Rev. vol. X. p. 547), we noticed with high praise, on its first publication, this singularly beautiful and excellent work. The new edition calls for the repetition of our encomiums, and with interest, inasmuch as all the old merits are enhanced by cognate novelties both of text and illustration. "In the present edition," says Mr. Wilson, "I have recorded the investigations of Mr. Bowman, on the minute anatomy of muscular fibre; of Mr. Nasmyth, on the development of the epithelium; of Mr. Curling, on the descent of the testis. I have also contributed some original researches which I have myself made on the minute structures of bone." Additions of importance are also made to the chapters on the ligaments, muscles, nervous system, organs of sense and viscera; and the number of woodcuts is increased from 150 to 167.

